# Comparison of DNA extraction kits and modification of DNA elution procedure for the quantitation of subdominant bacteria from piggery effluents with real-time PCR

**DOI:** 10.1002/mbo3.178

**Published:** 2014-05-18

**Authors:** Jérémy Desneux, Anne-Marie Pourcher

**Affiliations:** 1 Irstea-RennesRennes, France; 2 Université Européenne de BretagneRennes, France

**Keywords:** DNA extraction kit, elution procedure, *Lactobacillus amylovorus*, pig manure, qPCR, total bacteria

## Abstract

Four commercial DNA extraction kits and a minor modification in the DNA elution procedure were evaluated for the quantitation of bacteria in pig manure samples. The PowerSoil®, PowerFecal®, NucleoSpin® Soil kits and QIAamp® DNA Stool Mini kit were tested on raw manure samples and on lagoon effluents for their ability to quantify total bacteria and a subdominant bacteria specific of pig manure contamination: *Lactobacillus amylovorus*. The NucleoSpin® Soil kit (NS kit), and to a lesser extent the PowerFecal® kit were the most efficient methods. Regardless of the kit utilized, the modified elution procedure increased DNA yield in the lagoon effluent by a factor of 1.4 to 1.8. When tested on 10 piggery effluent samples, compared to the QIAamp kit, the NS kit combined with the modified elution step, increased by a factor up to 1.7 log_10_ the values of the concentration of *L. amylovorus*. Regardless of the type of manure, the best DNA quality and the highest concentrations of bacteria were obtained using the NS kit combined with the modification of the elution procedure. The method recommended here significantly improved quantitation of subdominant bacteria in manure.

## Introduction

Pig manure hosts a complex community of microorganisms that may vary during storage and during aerobic or anaerobic digestion. Two microbial aspects of manure treatment are the most frequently studied: (1) the health risks associated with pathogenic bacteria and (2) the role of the microorganisms in the transformation of the organic matter. Cultivation methods are usually used to evaluate the impact of manure treatment on pathogens and on indicator bacteria (fecal coliforms, enterococci) whereas molecular methods are more appropriate to identify the dominant microbial groups and shifts in the community composition (Leung and Topp 2001; Peu et al. 2006; Spence et al. 2008; Liu et al. 2009; Barret et al. 2012) and to detect or quantify specific markers of pig manure contamination (Ufnar et al. 2007; Marti et al. 2009, 2010; Mieszkin et al. 2009; Arthurson 2011). While methods based on culture are limited by the cultivability of bacteria, molecular methods may be biased by differential extraction of DNA from manure. Indeed, manure contains different substances including proteins, fulvic acids, and humic acids (Moller et al. 2004; Plaza et al. 2006), which can inhibit polymerase chain reaction (PCR). Moreover, aerobic and anaerobic digestion of pig manure produces effluents with different organic composition which can affect the efficiency of PCR. Most of the protocols for DNA extraction from manure samples are designed to isolate DNA from feces (Marti et al. 2009; Barkovskii et al. 2012) or from soil (Leung and Topp 2001; Ufnar et al. 2007; Spence et al. 2008; Mieszkin et al. 2009; Barkovskii et al. 2012). These DNA extraction procedures are essentially based either on mechanical and chemical lysis by chaotropic agents, detergents, and denaturing agents, or by a combination of heat, detergent, and mechanical action. Their efficiency in extracting DNA, which has been tested on feces (Tang et al. 2008), on sludge (Vanysacker et al. 2010) or on soil samples (Whitehouse and Hottel 2007; Dineen et al. 2010; Knauth et al. 2013), varies depending on the matrix analyzed. However, the efficiency of commercial kits for piggery effluents has not been fully explored. Furthermore, the quantitation bias is a major concern when PCR applications focus on the concentration of specific markers during the treatment process, as a low DNA extraction yield may lead to misinterpretation of the impact of the treatment. For such studies, highly efficient extraction of DNA from different type of manure is thus required.

The aims of this study were to compare the ability of four commercial DNA extraction kits to quantify total bacteria and, a specific pig manure marker, *Lactobacillus amylovorus*. In addition to the efficiency of the lysis the protocol used for DNA elution from the purification columns can contribute to differences in the recovery of DNA – we also compared procedures for DNA elution from the columns. The manure samples were selected for their different composition and for the treatment they underwent: raw manure stored in a tank, and treated manure stored in a lagoon after biological treatment and settling. The quantity and quality of the extracted DNA and quantitation of total bacteria and of *L. amylovorus* were compared to assess the performance of each kit.

## Materials and Methods

### Collection of manure samples

The efficiency of the DNA extraction kits and of the elution procedures were compared on two types of effluents collected from a piggery located in Brittany (France). Raw manure stored in a tank was centrifuged and further treated by aerobic digestion. Sludge was then separated from the liquid phase in a settling tank. The supernatant (liquid above the sludge) was removed and sent to a lagoon. Samples of raw manure and of lagoon effluent were collected. The characteristics of the raw manure and of the lagoon effluent were, respectively: dry matter, 43 and 7.6 g·L^−1^; organic matter, 27.4 and 2.0 g·L^−1^; total Kjeldahl nitrogen, 4.5 and 0.2 g·L^−1^; a pH of 7.8 and 8.6. Additional samples (eight raw manure samples and two lagoon effluents) were collected in eight other piggeries in order to compare the performance of the kits for the quantitation of bacteria. Samples were collected in a 1 L flask and stored at 4°C until analysis.

### Kit selection and DNA extraction

DNA extraction was performed using four commercial kits following the manufacturer's instructions. The kits, which used silica spin filter technology, were chosen for their easy and fast extraction methods. The following kits were evaluated: PowerSoil® DNA isolation and PowerFecal® kits (Mo Bio Laboratories Inc., Carlsbad, CA), QIAamp® DNA stool mini kit (Qiagen Inc., Valencia, CA), and NucleoSpin® Soil (Macherey-Nagel, Düren, Germany). For NucleoSpin® Soil, sample lysis was performed with the optional enhancer SX solution, as recommended by the manufacturer. In the rest of this article, the four kits are referred as PS, PF, QI, and NS, respectively.

To enable direct comparison of the kits, the same amount of starting material was used. DNA was extracted either from 250 mg of raw manure or from 250 mg of pellet after centrifugation of 90 mL of lagoon effluent at 16,000*g* for 30 min.

For each kit, two procedures were tested.

1. Procedure 1. DNA was eluted from the spin column with 100 *μ*L of the elution buffer following the manufacturer's instructions.
2. Procedure 2. The elution procedure was similar to that described for procedure 1, except that to optimize DNA yield, the eluent was split into four successive elutions, each performed with 25 *μ*L of elution buffer. The volume obtained after each centrifugation was pooled in one tube.

The kits performed with procedure 2 are further referred to as: PS_M_, PF_M_, QI_M_ NS_M_.

The samples of manure and lagoon effluent were extracted in triplicate using each kit. Extracted DNA was stored at −20°C.

### DNA yield and quality

The quality of the DNA extracts was estimated by running 3 *μ*L of purified DNA on a 1.5% agarose gel using 1× Tris-borate-Ethylenediaminetetraacetic acid (EDTA) buffer and a Lambda DNA HindIII marker (Promega, Madison, WI). Gels were visualized under UV light after gel staining with gel red (Interchim, Montluçon, France). The quantity (ng) and quality of extracted DNA were determined using a NanoDrop ND-1000 spectrophotometer (Nanodrop Technologies, Wilmington, DE).

### PCR

The presence of PCR inhibitors was assessed using a quantitative PCR assay targeting the bacterial 16S rDNA gene. DNA was amplified with universal eubacterial primers W18 and W02 (Table1).

**Table 1:** Sequence of the primers used in this study.

Name	Sequence	TM (°C)	Size amplicon (pb)	Reference
W18	5-′ GAGTTTGATCMTGGCTCAG-3′	50	1500	Godon et al. (1997)
W02	5-GNTACCTTGTTACGACTT-3′			Weisburg et al. (1991)
OTU171_RDA_F	5′TTCTGCCTTTTTGGGATCAA-3′	60	320	Konstantinov et al. (2005)
OTU171_RDA_R	5′CCTTGTTTATTCAAGTGGGTGA-3′			
1055F	5′-ATGGCTGTCGTCAGCT-3′	60	440	Ferris et al. (1996)
1392r	5′-ACGGGCGGTGTGTAC-3′			Amann et al. (1995)

Each PCR mix was prepared in a single tube. PCR reactions contained 1 *μ*L of template DNA undiluted or diluted 10 or 100-fold in water, 0.6 *μ*mol/L (each) forward and reverse primers, 2.5 U of Taq DNA polymerase (Sigma Aldrich, Saint Quentin Fallavier, France), 1× Taq DNA polymerase buffer 5× (Sigma Aldrich), deoxynucleoside triphosphates (Sigma Aldrich) at 2 mmol/L, 1.5 mmol/L MgCl_2_ (Sigma Aldrich). The reaction mixture was brought to a final volume of 25 *μ*L with water. PCR cycling was carried out using MJ Mini (Biorad, Paris, France). Bacterial 16S rDNA fragments were amplified in the following conditions: one cycle at 95°C for 2 min; 30 cycles of 94°C for 1 min, a denaturation step at 50°C for 1 min, one cycle at 72°C for 1 min; followed by a final extension at 72°C for 10 min. For each PCR reaction, a positive control (DNA from pure culture of *Listeria monocytogenes*) and a negative control (DNA free water) were used. The sizes of the amplification products were confirmed by agarose gel electrophoresis (1× Tris-borate-EDTA 1.5% [wt/vol] agarose) with 1.5 kb DNA BenchTop ladder (Promega). The PCR products were visualized under UV light after gel staining with gel red (Interchim). A PCR product with 1500 bp was considered a positive result.

### Quantitative PCR for *L. amylovorus* and total bacteria

*L. amylovorus* and total bacteria were quantified using primer pair OTU171_RDA_F/OTU171_RDA_R and 1055F/1392r (targeting 440pb of the 16S rDNA region), respectively (Table1). The reaction mixture consisted of 12.5 *μ*L of IQ SYBR Green Supermix (Bio-Rad), a 200 nmol/L concentration of each primer, 2 *μ*L of 1/10 diluted DNA, and 9.5 *μ*L of water to reach a final volume of 25 *μ*L. The PCR program for *L. amylovorus* is described in detail in Konstantinov et al. (2005). The PCR program for total bacteria included one cycle at 95°C for 10 min, 45 cycles at 95°C for 30 sec, one cycle at 60°C for 50 sec, and one at 72°C for 30 sec. PCR reaction was prepared using the automated pipetting system ep*Motion*® (Eppendorf, Le Pecq, France). PCR amplification was performed using a Bio-Rad CFX96 real-time PCR machine with Bio-Rad CFX Manager software, version 1.1 (Bio-Rad). All qPCR were performed in triplicate. Standard curves for *L. amylovorus* were constructed as described by Marti et al. (2010). A standard curve for total bacteria was generated as follows: The 16S rDNA fragments were cloned with StrataClone PCR Cloning Kit following the manufacturer's instruction (Agilent Technologies, Massy, France). Then plasmid extraction was performed with Qiagen plasmid mini prep (Qiagen) and purified with Wizard PCR clean Up (Promega, Wilmington, DE). The concentration of the initial plasmid solution was determined using Nanodrop. The standard curves were generated using 10-fold dilution of the plasmid solution containing 10^10^–10^2^ DNA copies·*μ*L^−1^. For each run, melting curve, and positive and negative controls were used.

### Statistical analysis

The amounts of extracted DNA and bacterial concentrations were analyzed using a repeated-measures one-way ANOVA followed by a Student–Newman–Keuls test in an all pairwise fashion. Bacterial concentrations were logarithmically transformed prior to analysis. All statistical tests were performed with XLSTAT 2010.4 (Addinsoft SARL, Paris, France).

## Results

The extraction efficiency of the four commercial kits, used according the manufacturer's instructions, was compared to that of the same kits with a modification of the final DNA elution step. ANOVA (Table2) revealed a significant effect of the DNA extraction method on the amount of DNA and on bacterial densities regardless of the type of manure used (raw manure or lagoon effluent).

**Table 2:** ANOVA results for DNA amount and bacterial concentrations as dependent variables and DNA kit extraction as predictor variables.

Variables	Matrix	Degrees of freedom	*F*-value	*P*
DNA	Manure	7	8.7	0.0002
	Lagoon	7	6.7	0.001
Total bacteria	Manure	7	205.1	<0.0001
	Lagoon	7	77.2	<0.0001
*Lactobacillus amylovorus*	Manure	7	7.9	0.0003
	Lagoon	7	54.8	<0.0001

### Total DNA yield

**Figure 1:**
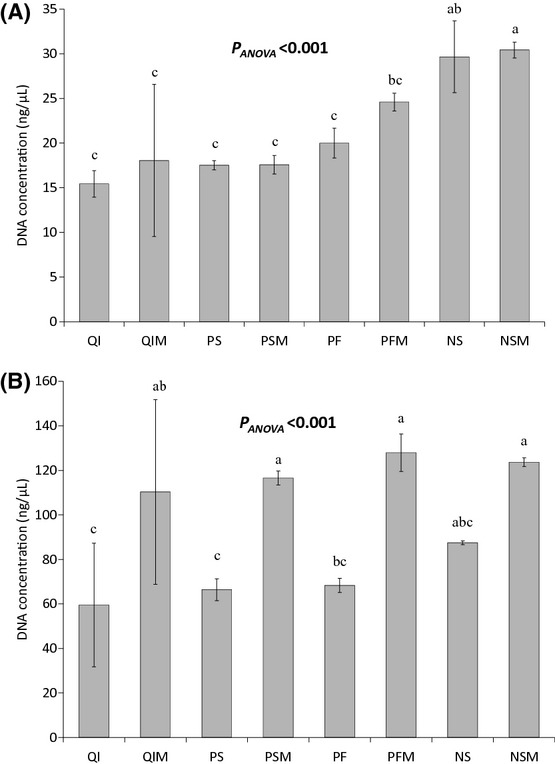
Concentration of DNA (ng·*μ*L^−1^) extracted by each kit per elution in manure (A) and lagoon effluent (B). The ANOVA *P* value is indicated in the figure. Letters above the bars indicate statistical grouping (Newman–Keuls test). When letters are shared among groups, they are not statistically different (*P *<* *0.05).

All four kits successfully evaluated DNA from manure and lagoon effluent but with varying efficiency (Fig.1). The NS_M_ kit produced two times more DNA than the Qi kit regardless of the matrix. DNA yields extracted from raw manure with the NS kit significantly differed from yields extracted with the Qi, PS and PF kits. The average concentrations of DNA extracted with the NS kit and the Qi kit were 29.7 ± 3.5 ng·*μ*L^−1^ and 15.4 ± 1.5 ng·*μ*L^−1^, respectively. It is noteworthy that with the Qi kit, the same amount of DNA was recovered after a second elution performed with 100 *μ*L of elution buffer on the column. With the other kits, the amount of DNA recovered in the second elution was very low and not significant compared to the amount obtained in the first elution (data not shown). In the case of the lagoon effluent, the difference in DNA yields between the NS kit and the others was less marked. However, the highest concentration of DNA in this matrix was also obtained with the NS kit (87.5 ± 0.8 ng·*μ*L^−1^). The modification of the elution procedure improved the yield of DNA extracted. The amounts of DNA extracted from manure were slightly higher using procedure 2, whereas the average DNA yield ratios from the lagoon effluent differed significantly with procedure 2 and procedure 1 and ranged from 1.4 to 1.8. The QI and QI_M_ kits showed low reproducibility for the lagoon effluent, and the Qi_M_ kit showed low reproducibility for manure, as indicated by the high variability of extraction efficiency between replicates (Fig.1).

### DNA purity

The level of DNA purity was determined by the *A*_260/280_ and *A*_260/230_ ratios. The Qi kit provided the lowest level of DNA purity (Table3). Regardless of the matrix, the *A*_260/280_ ratios of DNA extracted with procedures 1 and 2 were comparable, ranging between 1.72 and 1.84 and between 1.70 and 1.96, respectively (Table3), indicating that protein contamination was negligible. The *A*_260/230_ ratios with procedure 1 were low. They ranged from 0.87 to 1.21 for manure and from 1.42 to 1.77 for lagoon effluent. However, they were systematically increased with procedure 2. The extractions performed with the PF_M_ and NS_M_ kits had the highest *A*_260/230_ ratios, independently of the matrix (1.74 and 1.56 for manure, and 1.82 and 1.93 for lagoon effluent, respectively). The integrity of DNA extract was analyzed by agarose gel electrophoresis (Table3). Six of eight extractions produced undegraded DNA. The lowest yield was obtained when DNA was extracted from manure with the Qi kit. DNA extracted with the PS and PS_M_ kits produced a smear, indicating that DNA was probably degraded and thus unsuitable for bacterial quantitation. It is noteworthy that in preliminary test, a Qiagen protocol consisting of two elutions of 100 *μ*L was tested. However, this protocol did not enhance the yield and the purity of the DNA compared to the one step elution commonly recommended by the manufacturer (data not shown).To determine the relative sensitivity of the extraction kits, a dilution endpoint experiment using bacterial universal primers, was carried out. Extracts from samples were serially diluted and analyzed by PCR (Table3). Samples of manure and lagoon effluent consistently yielded amplification products in 1:10 dilutions except with the Qi kit. The optimum DNA yield – in terms of DNA quantity and quality – was obtained with the PF_M_ and NS_M_ kits.

**Table 3:** Comparison of DNA extraction kits on pig manure (Man) and lagoon effluent (Lag).

	DNA purity							
	*A*_260/280_		*A*_260/230_		DNA integrity^1^		Presence of PCR inhibitors^2^	
Kit	Man	Lag	Man	Lag	Man	Lag	Man	Lag
QI	1.72 ± 0.20	1.81 ± 0.07	0.87 ± 0.30	1.42 ± 0.30	±	**++**	Yes^3^	Yes^3^
QI_M_	1.79 ± 0.03	1.92 ± 0.03	1.40 ± 0.11	1.79 ± 0.16	++	**++**	No	No
PS	1.79 ± 0.11	1.87 ± 0.01	0.99 ± 0.26	1.80 ± 0.46	S	S	No	No
PS_M_	1.96 ± 0.27	1.88 ± 0.02	1.27 ± 0.28	1.83 ± 0.16	S	S	No	No
PF	1.83 ± 0.18	1.82 ± 0.02	1.17 ± 0.19	1.63 ± 0.04	++	**++**	No	No
PF_M_	1.70 ± 0.18	1.81 ± 0.06	1.74 ± 0.36	1.82 ± 0.12	++	**++**	No	No
NS	1.83 ± 0.15	1.84 ± 0.05	1.21 ± 0.52	1.77 ± 0.17	++	**++**	No	No
NS_M_	1.80 ± 0.06	1.87 ± 0.02	1.56 ± 0.28	1.93 ± 0.13	++	**+**	No	No

NS, NucleoSpin® Soil kit; PS, PowerSoil®, PF, PowerFecal^®;^ QI, QIAamp®.

1 ++, bright band; +, faint band; ±, very faint band; S, smear.

2 Observed using bacterial universal primers in the 1:10 dilution.

3 Presence of PCR inhibitor was not observed in the 1:100 dilution.

### Quantitation of total bacteria and *L. amylovorus*

A real-time PCR assay targeting the 16S rDNA or a genomic fragment of DNA for quantitation of total bacteria and *L. amylovorus*, respectively, was used to compare the efficiency of the four extraction kits and the two elution procedures from manure and lagoon effluent. Each kit was successful in quantifying *L. amylovorus* (Table4). However, regardless of the matrix, there was a significant difference in the amount of total bacteria and *L. amylovorus* measured. Depending on the kit, the amount of *L. amylovorus* ranged between 3.3 × 10^5^ and 2.6 × 10^6^ CFU eq·mL^−1^ in manure and between 1.7 × 10^2^ and 3.8 × 10^3^ CFU eq·mL^−1^ in lagoon effluent. In agreement with the extracted DNA yield, the highest amounts were obtained with the NS_M_ kit and the lowest with the PS and QI_M_ kits. The low quantitation of total bacteria and *L. amylovorus* with the PS kit is probably due to degraded DNA, underlining the importance of checking DNA integrity. Although the difference in the amount was not systematically significant, except with the Qi kit, the concentrations of total bacteria and *L. amylovorus* were always higher with procedure 2 than with procedure 1. To check their efficiency, the four kits were tested on supplementary samples of raw manure and lagoon effluents from eight other piggeries. For each kit, only the procedure corresponding to the highest concentrations of bacteria was chosen (procedure 1 for the Qi kit and procedure 2 for the three other kits). The concentrations of total bacteria and *L. amylovorus* obtained with the NS_M_ kit were systematically higher than those obtained with the QI and PS_M_ kits. They were similar to that obtained with the PF_M_ kit only in one manure sample and were higher for the other samples (data not shown). The average differences in calculated bacterial concentrations between the NS_M_ kit and each other kit ranged from 0.2 to 1.1 logarithmic units (Table5). As observed on the manure and the lagoon effluent tested in the first experiments, the lowest differences in concentrations were observed between the NS_M_ kit and the PF_M_ kit.

**Table 4:** Mean and standard deviation for concentrations of total bacteria and *Lactobacillus amylovorus* in manure and lagoon effluent after DNA extraction using four kits and two procedures of elution.

	Total bacteria (gene copies mL^−1^)		*L. amylovorus* (CFU eq·mL^−1^)	
Kit	Manure	Lagoon	Manure	Lagoon
QI	6.2 × 10^9^ (8 × 10^8^)^**C**^	6.8 × 10^7^ (1.3 × 10^7^)^**E**^	6.9 × 10^5^ (4 × 10^4^)^**BC**^	8.9 × 10^2^ (8 × 10^1^)^**D**^
QI_M_	5.7 × 10^9^ (2.2 × 10^8^)^**C**^	3.3 × 10^7^ (1.3 × 10^7^)^**F**^	4.2 × 10^5^ (1.9 × 10^5^)^**BC**^	1.4 × 10^3^ (1.4 × 10^2^)^**BC**^
PS	1.4 × 10^9^ (6.8 × 10^7^)^**E**^	1.4 × 10^7^ (6.1 × 10^4^)^**G**^	3.3 × 10^5^ (6.4 × 10^4^)^**C**^	1.7 × 10^2^ (5.1 × 10^1^)^**E**^
PS_M_	2.0 × 10^9^ (3.2 × 10^8^)^**D**^	4.9 × 10^7^ (1.2 × 10^7^)^**E**^	3.8 × 10^5^ (1.1 × 10^5^)^**BC**^	1.2 × 10^3^ (1.2 × 10^2^)^**CD**^
PF	6.5 × 10^9^ (6.1 × 10^8^)^**C**^	9.3 × 10^7^ (3.8 × 10^6^)^**CD**^	9.3 × 10^5^ (4.8 × 10^4^)^**BC**^	1.7 × 10^3^ (1.4 × 10^2^)^**BC**^
PF_M_	9.0 × 10^9^ (1.1 × 10^9^)^**B**^	1.8 × 10^8^ (1.1 × 10^7^)^**B**^	1.3 × 10^6^ (1.1 × 10^5^)^**AB**^	2.2 × 10^3^ (4.6 × 10^2^)^**B**^
NS	1.2 × 10^10^ (7.2 × 10^8^)^**A**^	1.1 × 10^8^ (1.0 × 10^7^)^**C**^	2.3 × 10^6^ (2.1 × 10^4^)^**A**^	1.9 × 10^3^ (8 × 10^2^)^**BC**^
NS_M_	1.3 × 10^10^ (6.1 × 10^8^)^**A**^	4.6 × 10^8^ (7.2 × 10^7^)^**A**^	2.6 × 10^6^ (4.8 × 10^4^)^**A**^	3.8 × 10^3^ (2.5 × 10^2^)^**A**^

Letters in bold indicate statistical groupings (Newman–Keuls test). When letters are identical among groups, they are not statistically different (*P *<* *0.05); NS, NucleoSpin® Soil kit; PS, PowerSoil®, PF, PowerFecal^®;^ QI, QIAamp®.

**Table 5:** Comparison of the efficiency of NS_M_ kit versus three kits in quantifying total bacteria and *Lactobacillus amylovorus* in pig manure samples and lagoon effluents.

		Differences of bacterial concentrations^1^	
Target (units)	Kit compared to NS_M_	Manure (8)^2^	Lagoon (2)
		Mean [min–max]	Mean [min–max]
Total bacteria (gene copies·mL^−1^)	QI	0.7 [0.5–1.3]	1.1 [0.7–1.5]
	PS_M_	0.7 [0.4–1.3]	0.4 [0.1–0.7]
	PF_M_	0.2 [0.0–0.6]	0.1 [0.1–0.1]
*L. amylovorus*(CFU eq·mL^−1^)	QI	0.7 [0.1–1.7]	−[0.8 to >1.0^3^]
	PS_M_	0.6 [0.1–1.3]	0.5 [0.2–0.7]
	PF_M_	0.5 [0.0–1.2]	0.3 [0.2–0.5]

Comparison was determined by subtracting the concentration of bacteria (expressed as log_10_) obtained with the NS_M_ kit from that obtained with each other kit. NS, NucleoSpin® Soil kit; PS, PowerSoil®, PF, PowerFecal^®;^ QI, QIAamp®.

1 Calculated as follows: log_10_ (concentration with the NS_M_ kit)- log_10_ (concentration with the Qi, PS_M_ or PF_M_ kit).

2 Number of samples.

3 *L. amylovorus* was not quantified with the QI kit.

## Discussion

DNA extraction is a critical step because it can affect both the quality and quantity of DNA extracted. Furthermore, an increase in DNA yield is important to ensure that the DNA sample is representative of the sample gene pool (Burgmann et al. 2001). The extraction yield mainly depends on the physical properties and chemical composition of the matrix (Zhou et al. 1996; Lakay et al. 2007; Yankson and Steck 2009). Indeed, extraction kits that are effective for feces or environmental matrices – such as activated sludge and soil – may not be appropriate for piggery effluents. The objective of the study was thus to compare the efficiency of commercial extraction kits for DNA quantitation of piggery effluents using real-time PCR. Because the chemical and physical properties of these types of effluents can vary depending on their management (storage or biological treatment), we evaluated two types of manure – raw manure and lagoon effluent, a by-product of the biological treatment – with different levels of dry matter (43 and 7.6 g·L^−1^). A modification of the elution step was tested to improve the extraction efficiency of the kits. The kits were selected on the basis of their widespread use for fecal and environmental samples (McGarvey et al. 2004; Grewal et al. 2006; Heuer and Smalla 2007; Ufnar et al. 2007; Ravva et al. 2011; Dalkmann et al. 2012; Lins et al. 2012), because they do not require special equipment and because they extract DNA rapidly (60–90 min).

Significant differences in DNA yields were observed among the kits. The Qi kit showed poor reproducibility and provided a lower yield than the other kits assessed in this study. This low yield may be due to incomplete cell lysis, as the Qi kit uses a chemical lysis procedure whereas the PS, PF and NS kits combine a chemical and a mechanical lysis by bead beating. Indeed, bead beating has been shown to improve DNA extraction efficiency of archae in samples of human feces (Salonen et al. 2010). The Qi kit, which appeared to be the least appropriate kit regardless of the level of organic matter of the effluent, was shown to be the most effective method for the extraction of DNA from human feces compared to four other commercial kits (McOrist et al. 2002). However, in agreement with our results, (Whitehouse and Hottel 2007) showed that this kit was less efficient for the extraction of DNA from pig feces.

The results of this study showed that the NS kit yielded the higher concentrations of DNA. This is in agreement with the study of Knauth et al. (2013), who compared three commercial kits using paddy soils and found that the NS kit provided the highest DNA extraction efficiency. The NS kit combines a specific buffer and a column to remove inhibitors which may be more efficient than the buffer used in the PS and PF kits.

The use of four successive elutions (procedure 2) improved the efficiency of DNA extraction, especially in the case of the lagoon effluent, leading to an increase of up to a factor 2 in DNA yield. The weaker improvement of procedure 2 on the DNA yield observed for raw manure may be explained by the amount of organic matter in the two matrices at the beginning of the extraction step. Centrifugation of the lagoon effluent produced a pellet containing around 25 times more organic matter than that in the manure analyzed. Procedure 2, which slows down the elution time, may be more efficient in eluting DNA in presence of higher concentrations of contaminants than procedure 1.

In addition to the amount of DNA extracted, the purity of the DNA, estimated by the ratios *A*_260/280_ and *A*_260/230_ and the integrity of the extracts checked by agarose gel electrophoresis, are also important parameters for gene amplification by PCR (von Wintzingerode et al. 1997). While the protein purity appeared to be satisfactory, the DNA extraction from the samples led to low *A*_260/230_ ratios, which reflected the co-extraction of contaminants absorbing at 230 nm such as residual phenol from nucleic acid extraction and humic and fulvic acids (Whitehouse and Hottel 2007; Techer et al. 2010). Interestingly, procedure 2 systematically led to an increase in *A*_260/230_ independently of the matrix, suggesting that the split of the elution volume improved the removal of contaminants from DNA.

Regardless of the extraction procedure and of the matrix, the PS kit showed a smear on agarose gel. This was not observed with the PF kit, which differed from the PS kit by an additional heat lysis step. To improve the quality of DNA extracted from biosolids, Taskin et al. (2011) modified the procedure recommended with the PS kit (1) by increasing the incubation time of the two buffers used for the cell lysis from 5 to 10 min, and (2) by heating the elution buffer to 60°C and then incubating it for 5 min at room temperature. These modifications were tested on the manure and the lagoon effluent in this study but did not improve the DNA yield (data not shown).

Given the high level of organic matter in piggery effluents, the extraction of DNA may lead to the co-extraction of humic acids and other contaminants that inhibit Taq polymerase activity (von Wintzingerode et al. 1997; Iacovacci et al. 2003). Only the Qi kit required a 100-fold dilution to be able to remove PCR inhibitors. The reagent used for removal of enzyme inhibitors contained in the Qi kit appeared to be less efficient than the specific buffers used by the three other kits and the column in the NS kit. Once again, procedure 2 reduced the PCR inhibitors with the Qi kit, confirming the importance of this extraction step not only in the DNA yield but also for the removal of the PCR inhibitors. Although QI has been used for environmental samples, it was initially developed for DNA extraction from stool. In contrast, NS and PS were developed for soil samples and PF is a modification of PS kit, adapted for feces. However, the inhibitors of PCR are mainly represented by humic acids in soil (von Wintzingerode et al. 1997) and by polysaccharides in stool (Agusti et al. 2010; Hall et al. 2013). The presence of humic and fulvic acids in manure may explain the lower efficiency of the QI compared to the three other kits. The efficiency of the kits was tested for the quantitation of total bacteria and of a marker of pig manure contamination, *L. amylovorus* whose levels, using the Qi kit, were close to or below the detection limit of qPCR in lagoon effluents (Marti et al. 2010). It clearly appears that except for the Qi kit, the modification in the elution procedure led to an increase in bacterial amounts. Furthermore, in accordance with the results of the DNA extraction, a higher quantitation of bacteria was obtained using the NS_M_ kit. When tested on 10 additional samples, compared with the results of the NS_M_ kit, the underestimation of the amount of *L. amylovorus* was substantial and reached up to 1.7 log_10_. It is noteworthy that the Qi kit which was previously used to extract DNA from manure and feces of cattle or pig (Lier et al. 2008; Tang et al. 2008; Liu et al. 2009; Marti et al. 2010; Alexander et al. 2011; Grewal et al.^2006^), gave the lowest amount of bacteria and failed to detect *L. amylovorus* in one lagoon effluent, suggesting that the occurrence of subdominant bacteria in piggery effluents may be underestimated with this kit. In conclusion, our results highlighted the fact that increasing the DNA extraction yield makes it possible to quantify subdominant bacteria in manure and lagoon effluents. The PS kit led to a degraded DNA and the Qi kit did not remove all the PCR inhibitors which led to significant underestimation of the concentrations of bacteria. By contrast, the NS kit and, to a lesser extent, the PF kit appeared to be more appropriate for these matrices. The modification of the final elution step increased the yield of DNA extracted and improved the quantitation of bacteria regardless of the target (total bacteria or less abundant specific bacteria). Therefore, to quantify bacteria in piggery effluents, we recommend the NS kit and the minor modification of the final elution step as described in Materials and Methods for commercial extraction kits using silica spin filter technology.

## References

[b1] Agusti G, Codony F, Fittipaldi M, Adrados B, Morato J (2010). Viability determination of *Helicobacter pylori* using propidium monoazide Quantitative PCR. Helicobacter.

[b2] Alexander TW, Yanke JL, Reuter T, Topp E, Read RR, Selinger BL (2011). Longitudinal characterization of antimicrobial resistance genes in feces shed from cattle fed different subtherapeutic antibiotics. BMC Microbiol.

[b3] Amann RI, Ludwig W, Schleifer KH (1995). Phylogenetic identification and in-situ detection of individual microbial-cells without cultivation. Microbiol. Rev.

[b4] Arthurson V (2011). Storage conditions and animal source influence the dominant bacterial community composition in animal manure. World J. Microbiol. Biotechnol.

[b5] Barkovskii AL, Manoylov KM, Bridges C (2012). Positive and negative selection towards tetracycline resistance genes in manure treatment lagoons. J. Appl. Microbiol.

[b6] Barret M, Gagnon N, Morissette B, Topp E, Kalmokoff M, Brooks SPJ (2012). Methanoculleus spp. as a biomarker of methanogenic activity in swine manure storage tanks. FEMS Microbiol. Ecol.

[b7] Burgmann H, Pesaro M, Widmer F, Zeyer J (2001). A strategy for optimizing quality and quantity of DNA extracted from soil. J. Microbiol. Methods.

[b8] Dalkmann P, Broszat M, Siebe C, Willaschek E, Sakinc T, Huebner J (2012). Accumulation of pharmaceuticals, Enterococcus, and resistance genes in soils irrigated with wastewater for zero to 100 years in central Mexico. PLoS One.

[b9] Dineen SM, Aranda R, Anders DL, Robertson JM (2010). An evaluation of commercial DNA extraction kits for the isolation of bacterial spore DNA from soil. J. Appl. Microbiol.

[b500] Ferris MJ, Muyzer G, Ward DM (1996). Denaturing gradient gel electrophoresis profiles of 16S rDNA-defined population inhabiting a hot spring microbial mat community. Appl. Environ. Microbiol.

[b10] Godon JJ, Zumstein E, Dabert P, Habouzit F, Moletta R (1997). Molecular microbial diversity of an anaerobic digestor as determined by small-subunit rDNA sequence analysis. Appl. Environ. Microbiol.

[b11] Grewal SK, Rajeev S, Sreevatsan S, Michel FC (2006). Persistence of Mycobacterium avium subsp paratuberculosis and other zoonotic pathogens during simulated composting, manure packing, and liquid storage of dairy manure. Appl. Environ. Microbiol.

[b12] Hall AT, Zovanyi AM, Christensen DR, Koehler JW, Minogue TD (2013). Evaluation of inhibitor-resistant real-time PCR methods for diagnostics in clinical and environmental samples. PLoS One.

[b13] Heuer H, Smalla K (2007). Manure and sulfadiazine synergistically increased bacterial antibiotic resistance in soil over at least two months. Environ. Microbiol.

[b14] Iacovacci G, Serafini A, Berti A, Lago G, Brinkman B, Carracedo A (2003). STR typing from human faeces: a modified DNA extraction method. Progress in forensic genetics 9.

[b15] Knauth S, Schmidt H, Tippkoetter R (2013). Comparison of commercial kits for the extraction of DNA from paddy soils. Lett. Appl. Microbiol.

[b16] Konstantinov SR, Smidt H, de Vos WM (2005). Representational difference analysis and real-time PCR for strain-specific quantification of *Lactobacillus sobrius* sp nov. Appl. Environ. Microbiol.

[b17] Lakay FM, Botha A, Prior BA (2007). Comparative analysis of environmental DNA extraction and purification methods from different humic acid-rich soils. J. Appl. Microbiol.

[b18] Leung K, Topp E (2001). Bacterial community dynamics in liquid swine manure during storage: molecular analysis using DGGE/PCR of 16S rDNA. FEMS Microbiol. Ecol.

[b19] Lier T, Johansen MV, Hjelmevoll SO, Vennervald BJ, Simonsen GS (2008). Real-time PCR for detection of low intensity Schistosoma japonicum infections in a pig model. Acta Trop.

[b20] Lins P, Schwarzenauer T, Reitschuler C, Wagner AO, Illmer P (2012). Methanogenic potential of formate in thermophilic anaerobic digestion. Waste Manage. Res.

[b21] Liu FH, Wang SB, Zhang JS, Zhang J, Yan X, Zhou HK (2009). The structure of the bacterial and archaeal community in a biogas digester as revealed by denaturing gradient gel electrophoresis and 16S rDNA sequencing analysis. J. Appl. Microbiol.

[b22] Marti R, Dabert P, Pourcher A-M (2009). Pig manure contamination marker selection based on the influence of biological treatment on the dominant fecal microbial groups. Appl. Environ. Microbiol.

[b23] Marti R, Dabert P, Ziebal C, Pourcher AM (2010). Evaluation of *Lactobacillus sobrius**L-amylovorus* as a new microbial marker of pig manure. Appl. Environ. Microbiol.

[b24] McGarvey JA, Miller WG, Sanchez S, Stanker L (2004). Identification of bacterial populations in dairy wastewaters by use of 16S rRNA gene sequences and other genetic markers. Appl. Environ. Microbiol.

[b25] McOrist AL, Jackson M, Bird AR (2002). A comparison of five methods for extraction of bacterial DNA from human faecal samples. J. Microbiol. Methods.

[b26] Mieszkin S, Furet J-P, Corthier G, Gourmelon M (2009). Estimation of pig fecal contamination in a river catchment by real-time PCR using two pig-specific bacteroidales 16S rRNA genetic markers. Appl. Environ. Microbiol.

[b27] Moller HB, Sommer SG, Ahring B (2004). Methane productivity of manure, straw and solid fractions of manure. Biomass Bioenergy.

[b28] Peu P, Brugere H, Pourcher AM, Kerouredan M, Godon JJ, Delgenes JP (2006). Dynamics of a pig slurry microbial community during anaerobic storage and management. Appl. Environ. Microbiol.

[b29] Plaza C, Hernandez D, Fernandez JM, Polo A (2006). Long-term effects of amendment with liquid swine manure on proton binding behavior of soil humic substances. Chemosphere.

[b30] Ravva SV, Sarreal CZ, Mandrell RE (2011). Bacterial communities in aerosols and manure samples from two different dairies in central and Sonoma valleys of California. PLoS One.

[b31] Salonen A, Nikkila J, Jalanka-Tuovinen J, Immonen O, Rajilic-Stojanovic M, Kekkonen RA (2010). Comparative analysis of fecal DNA extraction methods with phylogenetic microarray: Effective recovery of bacterial and archaeal DNA using mechanical cell lysis. J. Microbiol. Methods.

[b32] Spence C, Whitehead TR, Cotta MA (2008). Development and comparison of SYBR Green quantitative real-time PCR assays for detection and enumeration of sulfate-reducing bacteria in stored swine manure. J. Appl. Microbiol.

[b33] Tang J-N, Zeng Z-G, Wang H-N, Yang T, Zhang P-J, Li Y-L (2008). An effective method for isolation of DNA from pig faeces and comparison of five different methods. J. Microbiol. Methods.

[b34] Taskin B, Gozen AG, Duran M (2011). Selective quantification of viable *Escherichia coli* bacteria in biosolids by quantitative PCR with propidium monoazide modification. Appl. Environ. Microbiol.

[b35] Techer D, Martinez-Chois C, D'Innocenzo M, Laval-Gilly P, Bennasroune A, Foucaud L (2010). Novel perspectives to purify genomic DNA from high humic acid content and contaminated soils. Sep. Purif. Technol.

[b36] Ufnar JA, Ufnar DF, Wang SY, Ellender RD (2007). Development of a swine-specific fecal pollution marker based on host differences in methanogen mcrA genes. Appl. Environ. Microbiol.

[b37] Vanysacker L, Declerck SAJ, Hellemans B, De Meester L, Vankelecom I, Declerck P (2010). Bacterial community analysis of activated sludge: an evaluation of four commonly used DNA extraction methods. Appl. Microbiol. Biotechnol.

[b38] Weisburg WG, Barns SM, Pelletier DA, Lane DJ (1991). 16S ribosomal dna amplification for phylogenetic study. J. Bacteriol.

[b39] Whitehouse CA, Hottel HE (2007). Comparison of five commercial DNA extraction kits for the recovery of Francisella tularensis DNA from spiked soil samples. Mol. Cell. Probes.

[b40] von Wintzingerode F, Gobel UB, Stackebrandt E (1997). Determination of microbial diversity in environmental samples: pitfalls of PCR-based rRNA analysis. FEMS Microbiol. Rev.

[b41] Yankson KK, Steck TR (2009). Strategy for extracting DNA from clay soil and detecting a specific target sequence via selective enrichment and real-time (quantitative) PCR amplification. Appl. Environ. Microbiol.

[b42] Zhou JZ, Bruns MA, Tiedje JM (1996). DNA recovery from soils of diverse composition. Appl. Environ. Microbiol.

